# The caveolae-mediated sv40 entry pathway bypasses the golgi complex *en route *to the endoplasmic reticulum

**DOI:** 10.1186/1743-422X-2-38

**Published:** 2005-04-19

**Authors:** Leonard C Norkin, Dmitry Kuksin

**Affiliations:** 1 Department of Microbiology, University of Massachusetts – Amherst, MA 01003, USA

## Abstract

**Background:**

Simian virus 40 (SV40) enters cells via an atypical caveolae-mediated endocytic pathway, which delivers the virus to a new intermediary compartment, the caveosome. The virus then is believed to go directly from the caveosome to the endoplasmic reticulum. Cholera toxin likewise enters via caveolae and traffics to caveosomes. But, in contrast to SV40, cholera toxin is transported from caveosomes to the endoplasmic reticulum via the Golgi. For that reason, and because the caveosome and Golgi may have some common markers, we revisited the issue of whether SV40 might access the endoplasmic reticulum via the Golgi.

**Results:**

We confirmed our earlier finding that SV40 co localizes with the Golgi marker β-COP. However, we show that the virus does not co localize with the more discriminating Golgi markers, golgin 97 and BODIPY-ceramide.

**Conclusion:**

The caveolae-mediated SV40 entry pathway does not intersect the Golgi. SV40 is seen to co localize with β-COP because that protein is a marker for caveosomes as well as the Golgi. Moreover, these results are consistent with the likelihood that the caveosome is a sorting organelle. In addition, there are at least two distinct but related routes by which a ligand might traffic from the caveosome to the ER; one route involving transport through the Golgi, and another pathway that does not involve the Golgi.

## Background

Viruses commonly enter cells by receptor-mediated endocytosis; an entry pathway involving clathrin-coated pits and vesicles derived from them. These vesicles generally transport the virus to the endosomal/lysosomal compartment, where acidic conditions trigger virus disassembly and genome release [1].

Earlier experimental findings demonstrated that the entry pathway for simian virus 40 (SV40) might differ in important ways from the more common virus entry pathway. First, SV40 infection was found to be independent of the low pH of the endosomal/lysosomal compartment [2]. Second, electron microscopy studies showed that entering SV40 traffics to the endoplasmic reticulum (ER), rather than to endosomes [3]. More recently, SV40 was shown to enter cells via caveolae, rather than clathrin-coated pits [4,5]. Indeed, these were the first reports of a virus entering cells by means of caveolae. In addition, SV40 entry is signal-dependent, in contrast to the constitutive endocytosis of viruses that enter via clathrin-coated pits [6-8]. Finally, SV40 particles disassemble in the ER, rather than in the endosomal/lysosomal compartment [9].

Caveolae are small (70 to 100 nm) invaginations of the plasma membrane that are distinguished from clathrin-coated pits by their size, distinctive flask-like shape, and lack of a visible coat in thin sections. Expression of the protein caveolin-1 triggers the formation of caveolae in microdomains of the plasma membrane that are enriched in sphingolipids and cholesterol, and which are known as lipid rafts [10,11]. The cellular functions of caveolae are not yet entirely clear, but they have been implicated in organizing signal transduction pathways, and in sorting and trafficking through the endocytic and secretory pathways [12-14].

The endocytic trafficking of any ligand from the plasma membrane to the ER is most exceptional. Thus, it was important to ascertain the pathway taken by SV40. Cholera toxin (CT) provided a precedent for a possible SV40 entry pathway since it earlier had been shown to enter cells via caveolae, and also to traffic to the ER [15].

Interestingly, CT also can enter cells via clathrin-coated pits. Yet pharmacologically impairing the clathrin-mediated entry pathway had little effect on the toxic effects of CT. In contrast, selective inhibition of caveolae-mediated uptake prevented CT toxicity [15]. Importantly, productive SV40 infection likewise was prevented by pharmacologically impairing caveolae-mediated endocytosis, but not by blocking clathrin-mediated entry [4]. These experimental findings demonstrated that a caveolae-mediated entry pathway, rather than a clathrin-mediated pathway, is the physiologically relevant one for both the toxin and SV40. Moreover, these results also imply that the clathrin-coated pit-mediated pathway and the caveolae-mediated pathway do not mix or intersect at any point.

Importantly, CT traffics through the Golgi *en route *to the ER [16]. Transport of the toxin from the Golgi to the ER is mediated by the Golgi-to-ER retrieval pathway, which normally retrieves resident ER proteins that have escaped to the Golgi with the anterograde flux. In contrast, rather than trafficking to the Golgi, SV40 was seen to traffic to a new organelle, the caveosome. SV40 is then transported directly from the caveosome to the ER [17].

Little is known about caveosomes. They contain caveolin-1, but are non-acidic, and they do not contain markers for endosomes, lysosomes, or the ER. Importantly, caveosomes also do not contain the Golgi markers TGN46 or mannosidase II, implying that they are distinct from the Golgi [17].

Prior to the report that SV40 traffics to the ER via caveosomes [17], we set out to ask whether SV40, like CT, might traffic to the ER via the Golgi. We selected β-COP as our Golgi marker since this protein is best known as a component of the COPI coatamer complexes that mediate the retrograde retrieval pathway from the Golgi to the ER [18-21]. We found that SV40 indeed co localizes with β-COP before it enters the ER. However, in light of the report that SV40 traffics through caveosomes, rather than through the Golgi [17], we interpreted our findings and redirected our subsequent experiments as follows.

First, note that the recycling pathway from the Golgi to the ER is mediated by COPI coatamers that assemble on Golgi membranes [22-24]. Thus, since SV40, like CT, is transported from an intermediate compartment to the ER (from caveosomes in the case of SV40, and from the Golgi in the case of CT), we hypothesized that the SV40 pathway from caveosomes to the ER likewise might be mediated by COPI coatamers. This premise accounts both for the co localization of SV40 with β-COP [9], and the targeting of the virus to the ER.

Considering the above, we asked whether β-COP indeed might be present on caveosomes, and whether it might mediate trafficking from caveosomes to the ER. First, we demonstrated that β-COP in fact does co localize with caveolin-1 on an organelle that contains input SV40. Second, the simultaneous co localization of SV40 with both β-COP and caveolin-1 is seen before the virus appears in the ER [9]. Thus, the β-COP-containing intermediate organelle appears to be the caveosome [17]. Third, we and others reported that transport of SV40 from the intermediate organelle to the ER is blocked by the drug brefeldin A, which specifically inhibits the Ras-like GTPase ARF-1 that regulates assembly of COPI coat complexes [9,25]. Note that CT transport to the ER likewise is blocked by brefeldin A, as well as by microinjected antibodies against β-COP [23,26,27]. These experimental results confirm that SV40 indeed traffics through a caveolin-1-containing compartment, which most likely is the caveosome, en route to the ER. Moreover, they demonstrate that the caveosome, like the Golgi, is marked by β-COP. Finally, the pathway from the caveosome to the ER, like the retrograde pathway from the Golgi to the ER, is dependent on assembly of COPI coat complexes [9].

Now, for several reasons, we believe that it is important to revisit the question of whether SV40 is transported to the ER via the Golgi. Most importantly, there is the precedent provided by CT for a caveolae-mediated endocytic pathway that accesses the ER via the Golgi. This precedent becomes more compelling in view of the more recent discovery that CT traffics through a caveolin-1-containing "endosomal" compartment on its path to the Golgi [16]. Moreover, that compartment may well be identical to caveosomes, as demonstrated by the finding that when cells are allowed to simultaneously endocytose CT and SV40, these ligands are observed to co localize in caveolin-1-positive endosomes [16]. Yet CT then traffics to the Golgi en route to the ER, whereas SV40 is said to traffic directly from caveosomes to the ER. Finally, in most cultured cells caveolin-1 is seen in the Golgi, as well as at the cell surface.

In the current study, we sought to confirm our earlier finding that SV40 co localizes with β-COP [9]. In addition, we ask whether SV40 co localizes with two standard Golgi markers: golgin 97 and BODIPY-ceramide.

## Results and Discussion

**Figure 1:**
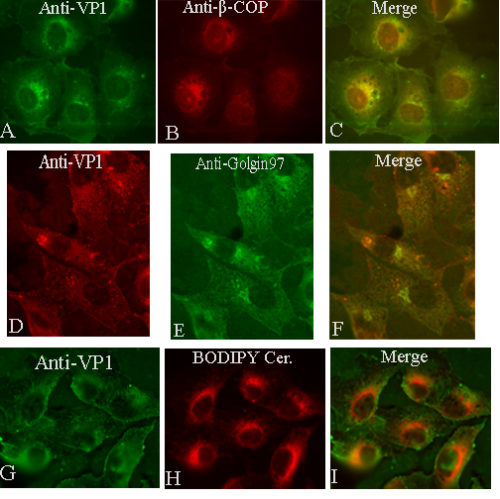
SV40 co localizes with β-COP (A, B, C, 3-hour sample), but not with the more stringent Golgi markers, golgin 97 (D, E, F, 5-hour sample) and BODIPY-ceramide (G, H, I, 3-hour sample).

In agreement with our earlier report [9], SV40 indeed co localized with β-COP at all three time points examined (3, 5, and 10 hours), although co localization was diminished by 10 hours (Figure 1A, B, and 1C, 3-hour sample shown). Regarding the latter observation, we demonstrated earlier that SV40 appears in the ER between five and ten hours, and most of the virus is in the ER by 10 hours [9].

In contrast to the early co localization of SV40 with β-COP, at no time was SV40 seen to co localize with Golgi markers golgin 97 (Figure 1D, E, and 1F, 5-hour sample shown) and BODIPY-ceramide (Figure 1G, H, and 1I, 3 hour sample shown).

The golgins in general are Golgi-localized proteins, characterized by an extensive coiled-coil structure throughout the entire molecule. The anti-human golgin 97 monoclonal antibodies used here recognize a 97 kD protein called golgin 97, a peripheral membrane protein that appears to be localized exclusively on the cytoplasmic face of the Golgi [28]. The exact function of golgin 97 is not known, although there is evidence that it may act to regulate transport between endosomes and the Golgi [29]. Fluorescent ceramide analogs, such as BODIPY-ceramide, are used extensively as selective stains for the Golgi [e.g., [30]]. They accumulate in this organelle, presumably because of its role in lipid biosynthesis and trafficking.

Indeed β-COP also is used as a Golgi marker. It preferentially associates with the lateral rims of the *cis *and medial Golgi cisternae, and the buds and vesicles derived from them. However, β-COP is not entirely specific to the Golgi since it also is associated with endosomal vesicles scattered throughout the cytoplasm [31,32]. Our own recent experimental results strongly implied that β-COP also is associated with caveolin-1-containing caveosomes [9]. Based upon the differential co localization of SV40 with β-COP, but not with the less promiscuous Golgi markers, golgin 97 and BODIPY-ceramide, we conclude that SV40 does not traffic through the Golgi *en route *to the ER.

Both SV40 and CT traffic from the plasma membrane to a common intermediate compartment, which appears to be the caveosome [16]. Since SV40 then traffics directly to the ER, whereas CT is first transported to the Golgi, the caveosome compartment would seem to have the ability to sort its different cargos for transport to different destinations. How this sorting might be achieved remains to be discovered. Caveolin-1 is not likely to play a role in this differential sorting of SV40 and CT, since that protein does not traffic with either ligand to its next destination [16,17]. Moreover, in cells lacking caveolin-1 expression and, therefore, caveolae, SV40 enters via lipid rafts, and still traffics to the ER via neutral organelles that resemble caveosomes, except that they do not contain caveolin-1 [33].

Understanding the determinants of these atypical trafficking pathways is fundamentally important since virtually all host ligands that are not internalized via clathrin-coated pits (e.g., sphingolipids, GPI-linked proteins) enter via sphingolipid and cholesterol-enriched membrane domains, and at least some traffic to a caveosome-like compartment, from which they then are sorted. This clathrin-independent mode of endocytosis likely enables these ligands to access sites that can not be accessed from the clathrin-dependent endocytic pathway.

SV40 binds to major histocompatibility complex (MHC) class I molecules at the cell surface [34,35]. However, MHC class I molecules do not internalize with the virus [36]. An interaction of SV40 with the ganglioside GM1 at the plasma membrane recently was shown to greatly enhance infectivity of the virus [37]. Perhaps SV40 uses GM1 as a co-receptor to deliver the virus into the cell. Interestingly, several bacterial toxins likewise use gangliosides for their cell surface receptors. In particular, CT binds to GM1 via its B subunit, and that interaction is necessary for the retrograde transport of CT via the Golgi to the ER [38]. It will be interesting to identify the factors which determine that SV40 takes a direct route from the caveosome to the ER.

## Conclusion

SV40 does not traffic through the Golgi *en route *from the cell surface to the ER. Based upon the current report and the earlier work of others regarding SV40 and cholera toxin [16], the caveosome appears to be an organelle able to sort its different cargos for transport to different destinations within the cell. Moreover, there are at least two distinct but related routes by which a ligand might traffic from the plasma membrane to the endoplasmic reticulum; one involving transport through the Golgi, and the other not involving the Golgi.

## Methods

### Cell cultures and infections

CV-1 cells (from the American Type Culture Collection) were seeded on 8 well Lab-Tek chamber slides (Nalge Nunc). SV40 was adsorbed to cells for 1 h at 4°C, at a multiplicity of infection of 50 to 100 plaque-forming units per cell. Cultures then were incubated at 37°C in Dulbecco modified Eagle medium plus 10% newborn calf serum (Atlanta Biologicals). At 3, 5, and 10 hours post infection, samples were washed five times in phosphate-buffered saline and fixed with 70% methanol at -20°C for 10 min.

### Confocal immunofluorescence microscopy

Confocal immunofluorescence microscopy was carried out using an epifluorescence Nikon E600light microscope. An ORCA-ER-cooled CCD camera (Hamamatsu) and OPENLAB software (Improvision) were used for all image acquisition and processing. Primary antibodies were monoclonal anti-β-COP antisera (Sigma), monoclonal anti-golgin 97 antisera (Molecular Probes), and our rabbit anti-SV40 antisera [9]. BODIPY (TR)-ceramide was from Molecular Probes. Secondary antibodies were fluorescein-conjugated goat anti-rabbit immunoglobulin G (IgG), Texas Red (TR)-conjugated goat anti-rabbit IgG, fluorescein-conjugated donkey anti-mouse IgG, and TR-conjugated donkey anti-mouse IgG (Jackson Laboratories, West Grove, PA). All antisera were diluted 1:100.

## Competing interests

The author(s) declare that they have no competing interests.

## Authors' contributions

LN conceived and supervised the study and drafted the manuscript. DK carried out all of the experimental work and data acquisition, taking important initiatives toward those ends. Both authors approved the final manuscript.
